# Case Commentary: Emergence of clinically relevant fidaxomicin-resistant *Clostridioides difficile* strains

**DOI:** 10.1128/aac.00482-26

**Published:** 2026-08-03

**Authors:** Kevin W. Garey, Julian G. Hurdle

**Affiliations:** 1 Department of Pharmacy Practice and Translational Research, University of Houston College of Pharmacy 15507, Houston, Texas, USA; 2 Center for Infectious and Inflammatory Diseases, Institute of Biosciences and Technology 26514, Houston, Texas, USA; 3 Department of Translational Medical Sciences, Naresh K. Vashisht College of Medicine, Texas A&M University 837698, Houston, Texas, USA; Houston Methodist Hospital and Weill Cornell Medical College, Houston, Texas, USA

**Keywords:** RNA polymerase, microbiome, antimicrobial resistance, *Clostridioides difficile *infection, antibiotics

## Abstract

*Clostridioides difficile* infection (CDI) has limited antibiotic treatment options, with fidaxomicin being the guideline-preferred antibiotic. C. E. Kaple, S. N. Redmond, J. L. Cadum, B. Hausman, et al. (Antimicrob Agents Chemother 0:e00130-26, 2026, https://doi.org/10.1128/aac.00130-26) describe a case of clinical failure in an elderly patient with CDI treated with a pulsed-taper fidaxomicin regimen, in which the MIC increased from 0.05 μg/mL at baseline to 32 μg/mL in isolates recovered during relapse. Relapsing symptoms were eventually resolved only after switching to oral vancomycin. This case, together with other recent clinical reports, may be the tip of the iceberg, signaling that fidaxomicin-resistant *C. difficile* can arise during treatment and contribute to clinical failure. It further challenges the assumption that high colonic drug concentrations mitigate resistance and underscores the need for improved surveillance and the development of clinically applicable molecular diagnostics to detect fidaxomicin resistance in CDI patient samples.

## COMMENTARY

Kaple et al. (1) report a case of an elderly patient in Cleveland, Ohio, with *Clostridioides difficile* infection (CDI) who relapsed while receiving pulsed-taper fidaxomicin. The fidaxomicin minimum inhibitory concentrations increased from 0.05 μg/mL against isolates at baseline to 32 μg/mL for isolates recovered during treatment failure. The isolates were genetically related, differing by only one to two single-nucleotide variants, supporting on-therapy emergence of resistance rather than reinfection with a new strain. The importance of this case extends beyond treatment failure in a single patient but rather adds to growing clinical evidence that fidaxomicin resistance can evolve during therapy and can be associated with clinical failure. Together, these findings highlight several challenges in CDI management: fidaxomicin resistance should no longer be dismissed as clinically irrelevant simply because stool drug concentrations are high; it may be underrecognized because routine CDI diagnostics do not include anaerobic susceptibility testing; and there is a need for research to guide the development of rapid molecular diagnostics to detect resistance in patient samples and to define the clinical impact of resistance within a pharmacomicrobiome context.

Fidaxomicin-resistant *C. difficile* was first identified during phase III clinical trials in a patient with recurrent disease, and subsequent treatment failures and resistant clinical isolates have since been reported in the United States, Europe, and Japan (2–4). Most recently, Redmond et al. (4), also in Cleveland, reported that 6% of isolates exhibited reduced fidaxomicin resistance, including three cases in which resistance emerged during treatment and was associated with clinical failure.

Fidaxomicin exerts its bactericidal activity by inhibiting transcription initiation through binding to *C. difficile* RNA polymerase (RNAP) inhibitor (5). Structural and genetic analyses of clinical isolates show that resistance-associated substitutions cluster at or near the fidaxomicin-RNAP binding site (6). To date, resistance mutations in clinical isolates have been limited to a small number of sites, including Val1143 and Asp814 in RpoB, and Arg89 and Arg326 in RpoC, suggesting fidaxomicin resistance may be genetically constrained, within a resistance-determining region. This presents a strong opportunity for developing targeted molecular diagnostics to rapidly detect resistance alleles and inform therapy. This may parallel rifamycin resistance, in which RNAP-targeting mutations arise within defined genetic regions and have enabled PCR-based diagnostics, such as the GeneXpert MTB/RIF assay targeting key RNAP mutations in *Mycobacterium tuberculosis* (7, 8).

Worryingly, resistance-associated RNAP mutations may not be deleterious, in terms of fitness cost, which limits clinical disease (4, 9). Consistent with this, the fidaxomicin-resistant *C. difficile* reported by Kaple et al. retained virulence *in vivo*, despite modest reductions in toxin production and sporulation observed *in vitro*. These findings raise concern that fidaxomicin-resistant strains may persist and be transmitted under increasing selection pressure from fidaxomicin use. Indeed, Redmond et al. (4) also reported the isolation of fidaxomicin-resistant isolates at baseline, indicating that these strains may be spreading. Moreover, extensive literature on rifamycin resistance in other bacterial pathogens highlights an additional layer of complexity for their pathogenesis and transmission, as RNAP mutations can remodel global transcription and influence virulence, persistence, and heterogeneous susceptibility to cell wall-active antibiotics, including β-lactams and daptomycin (10).

Data from phase III studies in Japan further complicate the interpretation of fidaxomicin resistance, as resistant isolates were recovered from patients classified as having achieved clinical cure. These secondary isolates, recovered within 4 weeks after treatment, were genetically related to baseline isolates. Similarly, Redmond et al. (4) identified fidaxomicin-resistant isolates at baseline that were not associated with treatment failure. Together, these findings highlight the complexity of interpreting the clinical impact of fidaxomicin resistance and suggest a potential role of host factors, including the microbiome in controlling CDI by these strains. It is likely that treatment outcomes for fidaxomicin-resistant strains may not follow a classical resistance paradigm. Because fidaxomicin is a narrow spectrum with limited effects on the broader microbiota, including *Bacteroides* species, the clinical behavior of resistant *C. difficile* may depend not only on the MIC shift itself but also on the capacity of the recovering microbiota to suppress resistant organisms to favor symptom resolution and clinical cure.

The clinical significance of fidaxomicin resistance is further complicated by uncertainties in its pharmacokinetics and pharmacodynamics. Although fidaxomicin achieves high colonic concentrations (~500–1,000 µg/g stool), it is poorly soluble, and only a fraction of the measured stool concentration may likely be microbiologically active (11). Nonetheless, these high colonic concentrations may create a false sense of security that elevated MICs may not lead to clinical failure, which reduces the perceived need for resistance testing. However, there are several problems with this expectation, as fidaxomicin is metabolized to the less potent compound OP1118, i.e., MICs increase 16-fold or more (12). This metabolism is thought to be directed by undefined intestinal esterases. These considerations raise several important pharmacokinetic/pharmacodynamic (PK/PD) questions relevant to clinical outcomes, including the extent to which drug metabolism contributes to mutant selection and treatment failure, the potential for inter-individual variability in fidaxomicin metabolism, and the role of host or microbial enzymes or non-biologic factors in this process. A better understanding of these factors will therefore be essential to define the clinical impact of fidaxomicin resistance.

In an era where there are few antibiotic treatment options for CDI, appreciating the clinical significance of emerging antimicrobial resistance to this limited set of antibiotics is an important first step in tackling the problem. The case report by Kaple et al. (1) is a great wake-up call to the infectious diseases, antimicrobial resistance, and drug discovery communities to consider *C. difficile* an antimicrobial-resistant priority pathogen.
